# *Truncus arteriosus communis*: report of three cases and review of literature

**DOI:** 10.4314/ahs.v18i1.19

**Published:** 2018-03

**Authors:** Henriette Poaty, Fanny Pelluard, Gwenaelle André, Brigitte Maugey-Laulom, Dominique Carles

**Affiliations:** 1 Histology-Embryology and Genetic Laboratory, Faculty of Health Sciences, BP 2672, Marien Ngouabi University, Brazzaville, Congo; 2 National Research Institute on Health Sciences, Brazzaville, Congo; 3 Department of Fetopathology, CHU Pellegrin, place Amélie Raba, 33076 Bordeaux cedex France; 4 Fetal Imaging Unit, Maternity, CHU Pellegrin, place Amélie Raba, 33076 Bordeaux, France

**Keywords:** Truncus arteriosus, conotruncal heart malformation, congenital heart defect, genetic etiologies

## Abstract

**Background:**

*Truncus arteriosus communis* (TAC) is a congenital heart defect in which the physiologic arterial common trunk was not divided into aorta and pulmonary artery trunk.

**Objectives:**

In this paper, we report on three observed cases from which we looked for (in conjunction with literature review) the different causes of TAC many of which have genetic origins.

**Methods:**

We collected three clinical files of fetuses having a TAC. Two of them were examinated after a medical termination of pregnancy motivated by severe cardiopathy. The malformation had been diagnosed based on different techniques: echocardiography, skeletal radiography, arteriography, fetal autopsy, karyotype and fluorescence in situ hybridization (FISH).

**Results:**

Imaging and fetopathological examination revealed the presence of TAC type 3 and 4 in the Van Praaghs classification. FISH analysis showed a 22q11.2 deletion in one fetus in favour of Digeorge syndrome. The karyotype analysis performed in two cases was normal.

**Conclusion:**

Truncus arteriosus is a rare pathology caused by numerous etiologies from which many of them have genetic origin. This malformation can be diagnosed early during prenatal period. Postmortem fetopathological examination allows a better diagnosis approach and eventually a genetic counseling in recurrent cases such as case of consanguinity.

## Introduction

Congenital malformations (CM) are commonly diagnosed in Africa, and the heart is one of the most affected organ in birth defects.^1^,^2^–^4^ The prevalence of congenital heart disease (CHD) worldwide is approximately 3.7 to 17.5 per 1,000 live births.^5^,^6^ In reality, it varies from one country to another, probably because of the difference in maternal risk factors, in the geographic environmental factors or in the inadequacy of diagnostic tools. In Australia, CHD prevalence ranges from 17.5 per 1,000 live births, while in Asia, CHD birth prevalence is higher than in Europe (9.3 per 1,000 live births vs. 8.2 per 1,000 live births).^6^,^7^ In Africa, CHD and its subtypes also vary among geographic areas.^8^,^9^,^10^ For instance, in Burkina Faso the prevalence of CHD is 9.8 per 1,000 live births with predominance of venticular septal defects (VSD), atrial septal defect (ASD), pulmonary stenosis (PS) and tetralogy of Fallot (TF).^9^ In Cameroon, the most common sub-types of CHD (in Yaoundé) are truncus arteriosus communis (TAC), transposition of great arteries (TGA) with ventricular septal defect (VSD) and Ebstein disease.^8^ In Congo Brazzaville, prevalence of CHD is 7.6 per 1,000 and the most frequently sub-types (revealed in two studies) are VSD, PS, ASD, patent ductus arteriosus (PDA) and TF.^1^,^10^ In general, the TAC subtype is not mentioned in many African CHD studies.

**Figure 1:**
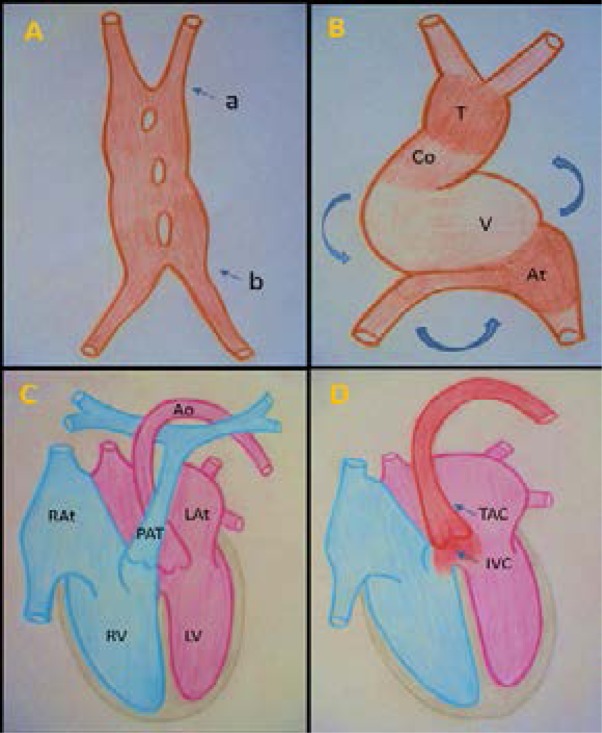
Formation of the heart and the persistent truncus arteriosus [15, 16, 19]. A) Fusion of two primordial endocardial tubes. To 21 days, formation of a cranial arterial extremity (a) and a caudal or sinus venosus extremity (b). B) Primitive cardiac tube. By day 22: presence of a single primitive ventricle (V), a single atrium (At) and a sinus venosus. Note that the cranial arterial extremity (become the conotruncal extremity) forms the physiological truncus (T) and the conus (Co). C) Normal heart. Note : two atria, right and left. Aorta (Ao) originating from left ventricle (LV) and a pulmonary artery trunk (PAT) arising from right ventricle (RV). Ao and PAT intersect each other. D) Truncus arteriosus (TAC). Large single arterial vessel arising from both ventricles above the ventricular septal defect (IVC).

The TAC is an embryonic conotruncal cardiac defect which consists in the persistence of the physiologic common arterial trunk (figure 1).

The two great arteries arising from the base of the heart (ascending aorta and pulmonary arterial trunk) do not differentiate, so there is occurrence of a single large vessel (with single arterial valve or truncal valve) that overhangs either a single ventricle or astride the two ventricles. The malformation is highly lethal, depending on the presence or absence of a pulmonary artery and associated extracardiac anomalies. The death without surgery occurs between two weeks and three months after birth, and the mortality rate is 85 % at one year.^11^ The TAC accounts for approximately 1 to 3 % of all congenital cardiac defects.^12^,^13^,^14^ However, this frequency may be underestimated for lack of good ultrasound imaging or due to the absence of post mortem fetal autopsy especially in developing countries. The purpose of this paper was to report on three cases of fetuses with TAC associated with extracardiac anomalies for two of them and to list (in conjunction with literature review) the etiologies of TAC especially those of genetic origins.

## Patients and methods

### Fetuses

We collected three clinical files of fetuses: two fetuses (F) of Congolese origin, M15 (F1) and 10F-0013 (F2) coming respectively from external consultation and our University collection; one fetus of French origin F11-048 (F3) originating from Department of Fetopathology of CHU Pellegrin (Bordeaux). Two of them (F1 and F3) were examined after a medical termination of pregnancy motivated by severe congenital heart defect. The fetal data is reported in Table 1.

**Table 1:** Fetuses' data

Fetuses	Motif of discovery	Age (W) and sex	Analyses	Macroscopic anomalies	Diagnosis
**1** (M15)	Fortuitous discovery (2^th^ trimester MU), MTP	22W, F	Echography, Karyotype (mother and fetus), FISH	Large TAC, agenesis of pulmonary artery trunk. Large high IVC	Isolated TAC, idiopathic cause
**2** (10F-001)	Death *in utero*, cesarean delivery	38W, F	Skeleton X-ray, Autopsy	Craniofacial dysmorphism. Skeletal extremity anomalies, large TAC riding on both ventricles, high IVC. Pulmonary arteries agenesis, dilatation of right cavities, two pulmonary lobes on right	Suspicion of trisomy 18
**3** (F11-048)	Intrauterine growth retardation, Cardiopathy, MTP	27W, F	Skeleton X-ray, Arteriography, Autopsy, Karyotype, FISH	Craniofacial dysmorphism, extremity anomalies. Large TAC with high IVC, right aortic arch, atresia of one pulmonary artery. Major aortopulmonary collaterals. Thymic hypoplasia, pseudocysts of adrenal cortex	Digeorge Syndrome

TAC: *truncus arteriosus communis*; IVC: interventricular communication; FISH: fluorescence in situ hybridization; W: weeks ; F: female sex; T: trimester; MU: morphology ultrasound; MTP: medical termination of pregnancy.

## Methods

For better diagnosis approach, three types of examinations were performed: (i) Imaging (including ultrasound, skeletal radiography and arteriography); (ii) fetal autopsy (in search of internal morphological abnormalities) on F2 and F3; and (iii) genetic analysis based on the karyotype and the FISH in two cases (F1 and F3).

Karyotype was performed on chorionic villus sampling and amniotic fluid. The conventional steps of blocking the cells (by adding colchicin), hypotonic shock, fixing with carnoy liquid and staining with Giemsa, were carried out after prior cell culture.

Fluorescence in situ hybridization (FISH) analysis was done in medical genetic laboratory of the Pellegrin University Hospital in order to identify the 22q11.2 microdeletion. The technique was performed by the use of TBX1-22qter cytocell probe (ref: LPU 014; TBX1, 22q11.21 in red and N85A3, 22q13.3 in green). The cell pellet was obtained from amniocytes culture.

## Results

The three fetuses had in common TAC visualized by radiography, ultrasound and/or by autopsy. FISH analysis confirmed a genetic etiology in one case.

**Figure 2:**
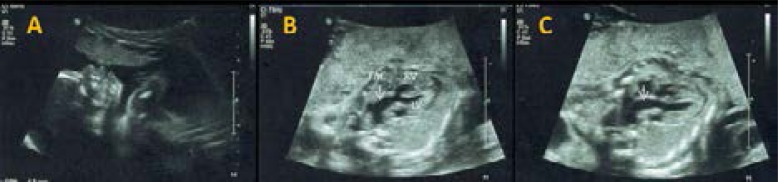
TAC ultrasound imaging. A) Fetal aspect (F1). B and C)Truncus arteriosus. Persistent common arterial trunk with agenesis of the pulmonary artery trunk and IVC.

Two-dimensional and three-dimensional fetal echocardiography performed in two cases: F1 (figure 2 B–C) and F3 allowed to easily diagnose the TAC and interventricular communication (IVC).

**Figure 4:**
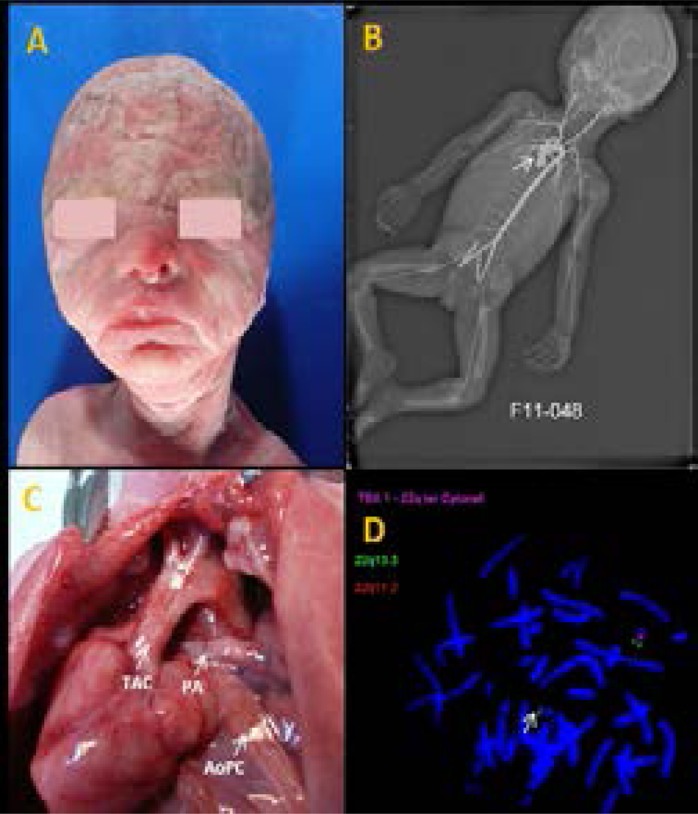
Macroscopic anomalies and FISH analysis. A) Craniofacial dysmorphy (F3). B) Arteriography: presence of common arterial trunk with right aortic arch. C) Autopsy image: common arterial trunk straddling the two ventricles, associated with atresia of one pulmonary artery arising from truncus (PA) and presence of aortopulmonary collateral arteries (AoPC). D) FISH analysis performed on metaphase mitosis showing: presence of a single signal red which indicates the microdeletion 22q11.2 in Di George syndrome.

Arteriography performed only in the F3 specified the TAC and visualized the right aortic arch (figure 4B).

**Figure 3:**
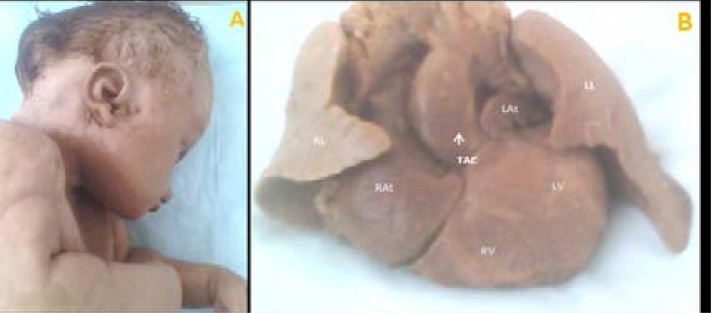
Extracardiac abnormalities associated to TAC. A) Facial anomalies (F2). B) Abnormal lung lobulation: presence of two pulmonary lobes on the right (RL). Large single arterial vessel in dextroposition on both ventricles (LV, RV) with agenesis of the pulmonary artery and dilatation of the right cavities (auricle and ventricle).

Fetal autopsy showed TAC type A4 in F2 (figure 3B) in the Van Praaghs classification and type A3 in F3 (figure 4 B–C) and visualized IVC.

Unfortunately karyotype analysis was not performed in case 2, due to the lack of fresh tissue while in the other two fetuses it was normal.

FISH analysis revealed 22q11.2 microdeletion in fetus 3 (figure 4D). On the other hand, it was found that no anomalies were in fetus 1. FISH results for fetus 2 were uninterpretable due to the bad preservation of tissues. All data on fetal outcomes are summarized in table 1.

## Discussion

The knowledge of the key aspects of heart development is important to understand where truncus arteriosus originates. The formation of the human heart begins at the third week of embryonic development (WED), at the stage of gastrulation and ends at eight weeks. It begins with the migration of two symmetrical waves of angiogenic cells on the primitive line and the formation of the mesenchyme of the cardiac field at the cranial extremity of the embryo, ahead of the pharyngeal region of the primitive intestine.^15^

Two primordial, lateral and symmetrical endocardial tubes appear within this field. These tubes are derived from the third embryonic layer: the mesoderm.^15^,^16^ At the 4^th^ WED during the embryonic plicature, the two tubes migrate at the thoracic level into a single primitive cardiac tube (PCT) (figure 1A), which has two extremities: (i) a cranial or arterial extremity (called conotruncal) formed of a conus and a truncus above the primitive ventricle (figure 1B); (ii) a caudal extremity or sinus venosus which receives three pairs of veins: vitellines, umbilicals, and cardinals.^16^ Subsequently, the PCT will draw a right-loop plicature (C-shaped) with the appearance of three angulations (figure 1B).^15^,^16^,^17^ The primordial cardiac cavities are at first a right system formed by a single right primitive atrium, a single arterial trunk (or Truncus arteriosus) which overhangs the single right primitive ventricle.^17^ The two cavities communicate through the atrioventricular canal.^16^,^17^ Therefore, initially the TAC is a physiological and transient embryonic step.

Between the 4^th^ and the 8^th^ WED, the PCT undergoes rapidly modifications which give rise to the four normal cardiac cavities and to the normal situs of the heart (figure 1C).^15^ From the 5^th^ WED at the level of the physiological arterial trunk, we have a migration of the conus and a conotruncal rotation.^15^,^16^,^17^ The migration of the conus allows its insertion between the two atrioventricular orifices coming from the primitive atrioventricular canal, in position of a transient physiological dextroposition. The conus gives rise to the infundibular septum and the ventricular septum in its upper part.^17^

The great vessels of the heart basis which originate from the primitive ventricle are in the beginning parallel and in physiological transposition: the aorta is forward and right and the pulmonary artery trunk is on the left. The reverse conotrunc rotation carries the pulmonary artery forward, right and up; the aorta is found on left and back. The two great cardiac vessels intersect each other and align themselves on their ventricles.^17^

A defect in the conal migration and the conotruncal rotation will lead to a defect in the conotruncal septation at the origin of the persistent truncus arteriosus communis. The latter is usually accompanied by IVC as observed in our three fetuses (table 1). Although TAC without ventricular septal defect exists, it is rare.^18^,^23^,^29^,^30^ TAC is also described in 5p13.2 deletion (Cat Cry syndrome), in 7q deletion, in trisomy 8 by mosaic and in duplication 8q.^14^,^24^,^31^ The translocations, the inversions, or deletions of the chromosome X are also concerned.

Truncus arteriosus may also be a warning sign of several genetic syndromes that generally represent the third cause of TAC (18.75% of CTC).^22^ 22q11.2 microdeletion (involving Digeorge's syndrome and related forms) found in one of our fetuses (figure 4D) is one of the frequent causes of TAC.^13^,^18^ It accounts 15.6% of the TAC, i.e. 2.3% of all CHD in the series of Fredouille et al.^22^ Usually, the prevalence of 22q11.2 microdeletion in the TAC is estimated at 20-50% and the most pointed gene is the transcription factor TBX1(OMIN # 217095).^21^,^14^,^32^,^33^ The other genetic syndromes labeled, indexed in the genesis of the TAC are: IVEMARK syndrome, FRYNS syndrome, Carpentier syndrome, Ellis Van Creveldt syndrome, Cornelia De Lange syndrome or Lempitz-Opitz syndrome, Meckel-Gruber syndrome.^29^

The mutations of the following genes: NKX2-5 mapped on 5q35.1 and NKX2-6 in 8p21.2 l band can also generate isolated family TACs (OMIM #217095).^13^ The malformation can also due to a deleterious GATA6 mutation present in 18q11.2, which is accompanied by agenesis or pancreatic hypoplasia causing neonatal diabetes or GDF1 mutation located in 19p13.1 locus (OMIM #217095).^34^ Other indexed candidate genes are PRKD1, NRP1 and PRDM1.^13^,^35^

In the associations: TAC may also be part of a VACTERL, combination of vertebral, anal, cardiac, tracheo-esophageal, renal, and limb abnormalities. CHARGE in which a mutation of the CHD7 gene is observed in two thirds of patients is also concerned.^36^ The latter condition includes coloboma, heart defect, atresia choanae, retarded growth and development, genital hypoplasia and ear anomalies.^14^ Various maternal risk factors indexed in literature can also induce the TAC. Others factors that can increase the risk for conotruncal defects in fetuses can be: infections in utero such as maternal rubella; metabolic disorders such as maternal diabetes mellitus type 1 and 2; chronic maternal disease: hypertension, obesity.^26^,^37^–^40^ Persistent common arterial trunk is also observed in populations having a high parental consanguinity rate.^13^,^41^

The following factors occurring during gestation are also implicated: stress, alcohol, cigarettes smoking, abusive coffee, retinoic acid, maternal drug consumptions for example valproic acid (anti-epileptic drug used in maternal epilepsy).^29^,^37^ Maternal environmental exposures before conception and in early gestation to minerals, hydrocarbons and oil derivatives can also induce conotruncal heart defect.^2^ But, all these maternal risk factors are not specific to the TAC. They can cause other lesions listed in table 2.^42^–^47^

**Table 2:** Comparison of TAC percentage with others common subtypes of CHD in diverse studies.

Countries	PCHD (‰)	VSD (%)	TF (%)	ASD (%)	PS (%)	PDA (%)	TGA (%)	TAC (%)	Authors
**Congo** **Brazzaville**	7.6	30.1	10.1	20.3	29.8	11.7	0.9	1.6	Pemba et al. 2016 [10]
**Burkina** **Faso**	6.1	27.2	9.1	10.6	1.5	1.5	1.5	6	Tougouma et al. 2016 [42]
	9.8	28.26	9.42	23.19	19.57	6.52	U	U	Kinda et al. 2015 [9]
**Nigeria**	14.4	27.1	8.4	2.5	0.9	14.5	3.6	0.9	Otaigbe et al. 2014[2]
**Senegal**	8	24.4	9.8	3.7	1.2	7.3	1.2	U	Ngouala et al. [43]
**Rwanda**	(**)	27.08	8.3	8.3	4.16	29.16	U	4.16	Teteli et al. 2014 [44]
**Tunisia**	6.8	31	6.2	12.9	2.7	U	2.7	-	Abid et al. 2014 [45]
**India**	8.07	43.9	1.8	7.3	1.2	4.3	4.3	1.2	Saxena et al. 2016 [46]
**Mali**	(*)	35.29	9.92	31.37	U	1.78	11.76	U	Hava Daou 2008 [47]

PCHD: Prevalence of CHD ; VSD: ventricular septal defect; ASD: atrial septal defect ; TF: Tetralogy of Fallot ; PS: pulmonary stenosis ; PDA : patent ductus arteriosus ; TGA: transposition of the great arteries ; TAC: truncus arteriosus communis ; U: unspecified, *study from 51 CHD cases ; **study from 48 CHD cases

## Conclusion

Previous data have shown that the origin of TAC is varied, particularly in genetic origins. The good fetal echocardiography in the first trimester of gestation (since 12 weeks of embryonic development) allows an early diagnosis and guides the behavior very early because some types can be surgically repaired. Postmortem fetopathological examination allows for better delineation of anomalies and can lead to genetic counseling in recurrent cases such as in parental consanguinity.

What is already known on this topic shows TAC is a rare, serious congenital heart defect of various causes and it can be detected in prenatal period by routine ultrasound screening. Interestingly, our paper reports uncommon type A3 and A4 of TAC, lists the TAC associated genetic defects and highlights the fetopathological examination as a value tool in positive diagnosis of TAC. The discipline, often mistakenly regarded as a luxury medicine in Africa, now needs to be developed in the South countries as the incidence of congenital malformations is increasing.

## Figures and Tables

**Table 3:** Collett and Edwards' Classification [11, 14]

Types	Description
**I**	Truncus arteriosus with hypoagenesis of pulmonary arterial trunk (arising back the aorta) and bifurcating in two pulmonary arteries right and left
**II**	Truncus arteriosus with the two pulmonary arteries arising separately from the posterior portion of the truncus
**III**	Truncus arteriosus with the two pulmonary arteries arising separately from the lateral walls of the truncus
**IV**	Truncus arteriosus with agenesis of pulmonary arterial trunk (and absence of pulmonary arteries), strongly supplemented by aortopulmonary collaterals
